# Pore structure modified diatomite-supported PEG composites for thermal energy storage

**DOI:** 10.1038/srep32392

**Published:** 2016-09-01

**Authors:** Tingting Qian, Jinhong Li, Yong Deng

**Affiliations:** 1 School of Materials Science and Technology, Beijing Key Laboratory of Materials Utilization of Nonmetallic Minerals and Solid Wastes, National Laboratory of Mineral Materials, China University of Geosciences (Beijing), Beijing, 100083, China

## Abstract

A series of novel composite phase change materials (PCMs) were tailored by blending PEG and five kinds of diatomite via a vacuum impregnation method. To enlarge its pore size and specific surface area, different modification approaches including calcination, acid treatment, alkali leaching and nano-silica decoration on the microstructure of diatomite were outlined. Among them, 8 min of 5 wt% NaOH dissolution at 70 °C has been proven to be the most effective and facile. While PEG melted during phase transformation, the maximum load of PEG could reach 70 wt.%, which was 46% higher than that of the raw diatomite. The apparent activation energy of PEG in the composite was 1031.85 kJ·mol^−1^, which was twice higher than that of the pristine PEG. Moreover, using the nano-silica decorated diatomite as carrier, the maximum PEG load was 66 wt%. The composite PCM was stable in terms of thermal and chemical manners even after 200 cycles of melting and freezing. All results indicated that the obtained composite PCMs were promising candidate materials for building applications due to its large latent heat, suitable phase change temperature, excellent chemical compatibility, improved supercooling extent, high thermal stability and long-term reliability.

In the past decades, renewable energy utilization has been the word top due to the environment issues and the depletion and high cost of fossil fuel. Solar energy has to be the most concerned one in the world because it is inexhaustible, abundant and clean^1^. However, the fluctuation of solar radiation, which is resulted from the climatic and seasonal variation, makes thermal energy storage system indispensable in solar thermal energy applications^2^. The technique of latent heat storage employing phase change materials (PCMs) is the most concerned. Phase change materials (PCMs) are latent heat energy storage materials that possess the competitive advantages of high energy storage density, isothermal operating characteristics, and smaller temperature variation^3^,^4^. Thus, they have triggered the interest of academic and industrial application in storing thermal energy collected from solar radiation.

PEG was the most widely studied PCM due to its suitable phase change temperatures and high thermal storage capacity, which can be easily tuned by varying molecular weight. In our study, PEG (Mw = 4000) was selected whose melting temperature is around 60 °C. Moreover, PEG possesses congruent melting behavior, excellent chemical and thermal stabilities, quite low vapor pressure, non-toxicity, and competitive cost^5^,^6^. However, there are still several insurmountable defects for applications of PEG PCMs in traditional manners. For example, it is difficult to handle the materials during the phase change from solid to liquid. Low thermal conductivity and high degree of supercooling lead to the hysteresis of thermal response. In addition, the PEG PCMs also have an interfacial combination problem with surrounding materials^7^. Such drawbacks may degrade the performance of energy storage and thermal regulation during the melting and freezing cycles and restrict their final applications. As far as the practical application is concerned, it is worthwhile to transform solid–liquid PCMs into novel shape-stabilized PCMs (ss-PCMs). Here, the powder–like and organic–inorganic ss-PCM can maintain its solid shape even when the thermal energy storage substance is changed from solid to liquid state^8^,^9^. That is, the supporting material can make liquid PEG convenient to control and prevent PEG from adverse interactions with surrounding materials and environment.

Various materials have been utilized to prepare shape-stabilized PCMs^10^,^11^,^12^. Diatomite, a type of natural amorphous siliceous mineral from geological deposits, possesses a variety of unique properties including the highly porous structure (80–90%), excellent absorption capacity, low density, chemical inertness, and relatively low price^13^,^14^,^15^,^16^. With these advantages, diatomite can be seen as a sort of feasible low-weight carrier material of PCMs for energy storage in buildings. For example, Karaman *et al*.^6^ prepared a novel shape-stabilized composite PCM by incorporating polyethylene glycol (PEG) into diatomite and 50 wt% PEG was retained into the pores of RMS without any seepage. With diatomite, Li *et al*.^17^ prepared several kinds of binary fatty acid/diatomite shape-stabilized phase change materials via a facile fusion adsorption method. The latent heat of the composite decreased to 57% of that of capric–lauric acid PCMs and the phase-transition temperature rose slightly from 16.36 to 16.74 °C. Xu *et al*.^18^ prepared the paraffin/diatomite ss-PCMs using the direct impregnation method. The results indicated that paraffin percentage is 47.4 wt%. Li *et al*.^19^ employed three grades of diatomite particles to produce phase change material composites by absorbing paraffin into the pores of diatomite particles. The maximum absorption ratio of paraffin is 50%, 38% and 35%, respectively, by mass percentage of paraffin in the composite. Investigation results presented by Nomura *et al*.^20^ and Sarı *et al*.^21^ also demonstrated that diatomite was an ideal supporting matrix for PCM. However, the diatomite particles employed in these PCM composites were raw materials without further modification, resulting in the low adsorption capacity less than 50%, which will in turn reduce the energy-storing density. Although the leakage problem is usually circumvented by introducing shape stabilization support, this is inevitably lead to the reduction in energy storage density. In order to enhance the thermal storage capacity, considerable efforts have been devoted to the development of novel ss-PCMs with high PCM load^14^.

However, diatomite has its shortcoming that the pores of raw materials are commonly blocked by several types of impurities, thereby decreasing its microporosity, filtration efficiency, and commercial applicability. Hence, raw diatomite needs to be purified before its commercial utilization^22^,^23^,^24^. Until now, different methods have been employed, including calcination, acid leaching, a scrubbing method, high-speed shear, and ultrasonication^14^,^22^,^25^. However, with these methods, inefficiency (scrubbing method), destruction of the diatomite structure (high-speed shear) and high processing cost (ultrasonication) compromised the results^26^. Much attention has been payed to the modification process that is low-cost, easy to operate and environment friendly.

Currently, modified diatomite-supported PEG composite PCMs has been little documented. In our study, diatomite was firstly treated by calcination, acid treatment, alkali leaching and nano-silica decoration. PEG/modified-diatomite composite PCMs were systematically fabricated; then characterization and properties of these composite PCMs were determined. The prepared PEG/diatomite ss-PCM will be a potential candidate for the application in the fields of building envelopes in the continuous hot summer, whose temperature is often as high as 50~70 °C.

## Results

### Characterization of modified diatomite

**Figure 1:**
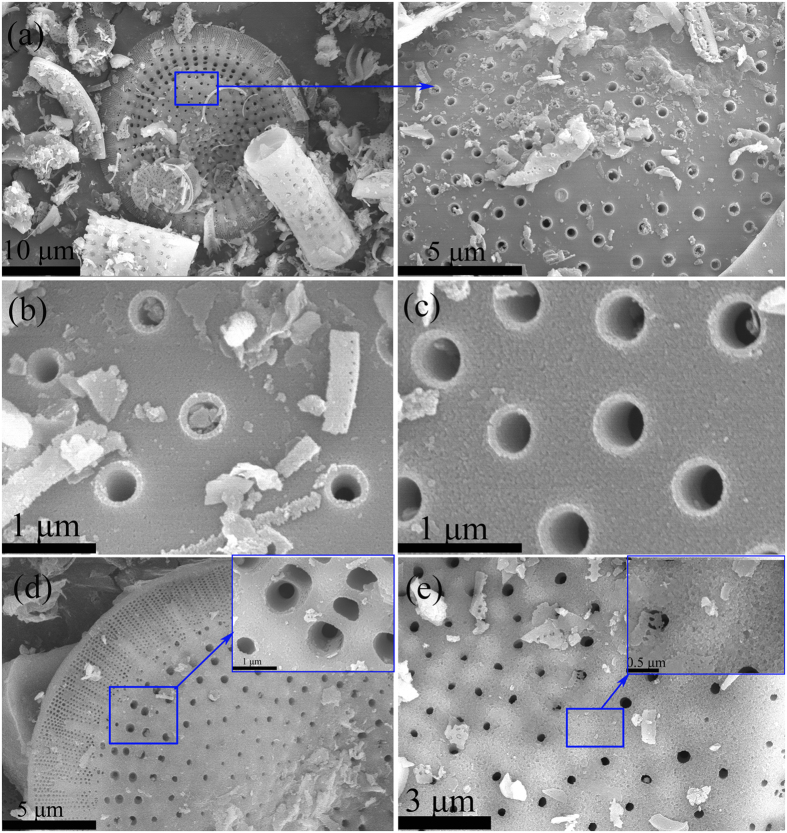
SEM images of: (**a**) raw diatomite (RD); (**b–e**) modified diatomites (RD-1, RD-2, RD-3, and RD-4).

Figure 1 demonstrates the microstructures of raw diatomite and the modified samples. From Fig. 1(a), diatomite powder is mainly composed of disc-like and cylindrical structures and numerous pores can be clearly seen, indicating the high porosity and large specific surface area of diatomite as expected. However, there were a lot of impurities on the surface of the raw diatomite. Moreover, most of the pores on the surface of raw diatomite were blocked by visible impurities. As seen from Fig. 1(b), after thermal treatment, no obvious improvement was found. In Fig. 1(c), it is clearly seen that there were less impurities and that the pores on the surface of diatomite became more obvious. In Fig. 1(d), the morphology of the porous structure is basically retained after alkali leaching. And the clogged pores were dredged in various degrees. For PEG PCM carrier, it is a good phenomenon because more PEG can be adsorbed in these pores. In Fig. 1(e), it is worth noting that the nano-silica particles were observed on the surface of diatomite and the porous structure of diatomite remained clearly visible.

**Figure 2:**
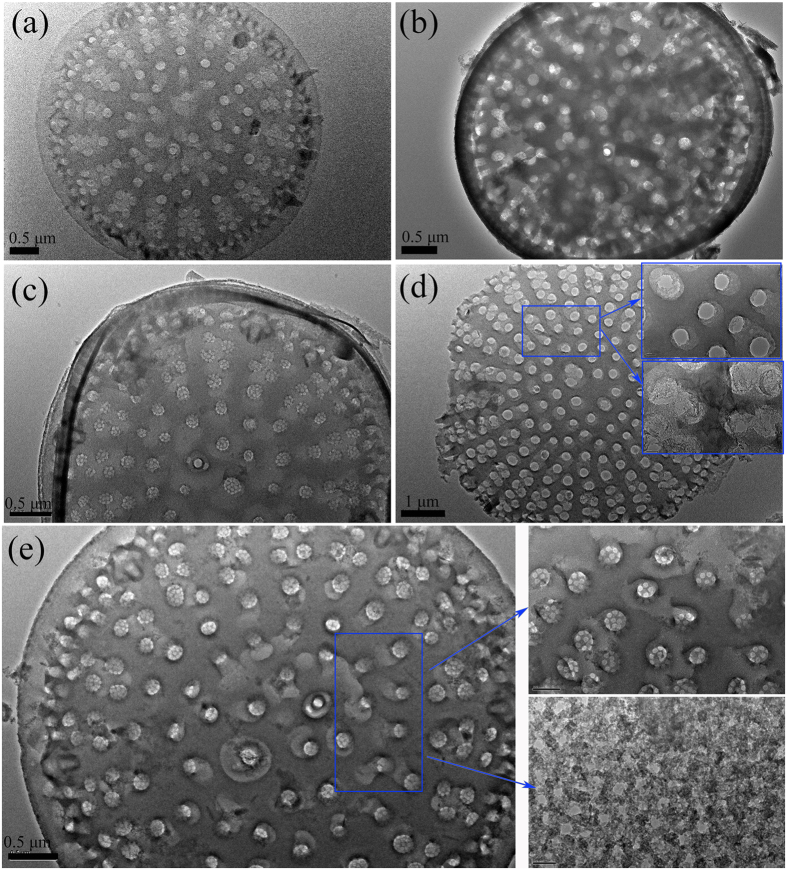
TEM images of: (**a**) raw diatomite (RD); (**b–e**) modified diatomites (RD-1, RD-2, RD-3, and RD-4).

In order to confirm the observations obtained from SEM and observe the morphology on the pores structure of the modified diatomite more precisely, TEM was also applied in this study (Fig. 2). From Fig. 2(a), one can see numerous macropores in the middle region and ordered mesopores in the peripheral area of the diatomite disk, which are in good agreement with the SEM observations. However, most macropores are blocked. After thermal treatment, the organic substances existing in pores of diatomite were volatilized, which resulted in the increase of specific surface area of diatomite. From Fig. 2(b), some of the pores can be clearly seen. In Fig. 2(c), after the treatment with 20 wt.% sulfuric acid, plenty of pores can be clearly seen. Due to the reaction between metallic oxides and sulfuric acid, the original clogged pores were opened and the pore edges were enlarged gradually. The result indicates that metallic oxide is the main stuff which clogs the pore. As SiO_2_ is the main component of diatomite, treatment with sodium hydroxide leads to the formation of soluble silicates SiO_3_^2−^, resulting in the creation of larger pores, flaws, cracks and a larger surface areas^27^. From Fig. 2(d), after treatment with the sodium hydroxide solution, the multi-pore structure of diatomite basically remained intact and the pores on the surface became larger. Interestingly, some spongy-like pores can be easily found, which will benefit for PEG adsorption. However, as shown in Fig. S1, the overall morphology of diatomite was getting destroyed when the leaching time exceeds 8 min, which is undesirable for the PEG carrier. Therefore, we concluded that alkali leaching is an effective modification approach on diatomite to dredge the pores and the optimum time is 8 min. Figure 2(e) illustrates the TEM image of the nano-silica/diatomite samples. From Fig. 2(e), nano-silica particles were loaded onto the surface of diatomite instead of into the pores. The detailed morphologies of nanoparticles on the surface is also shown in the inset picture and the particle size is around 30~50 nm. Those nano-silica particles enhance the exterior roughness of diatomite, enlarging its specific surface area and improve the PEG adsorption capacity. However, it is clearly seen that the pores on the diatomite surface were seriously obstructed when the concentration of silica precursor (Na_2_SiO_3_) rises above 0.03 mol·L^−1^ (see in Fig. S2).

The main chemical compositions of raw and modified diatomite are listed in Table 1. As seen in this table, RD is mainly composed of 86.82 wt% SiO_2_. After the purification, the contents of Al_2_O_3_ and TFe_2_O_3_ are significantly decreased, indicating that the thermal and acid treatments are efficient. Besides, after the alkali leaching and nano-silica decoration, the SiO_2_ contents are respectively decreased by 6% and increased by 1.3%.

**Figure 3:**
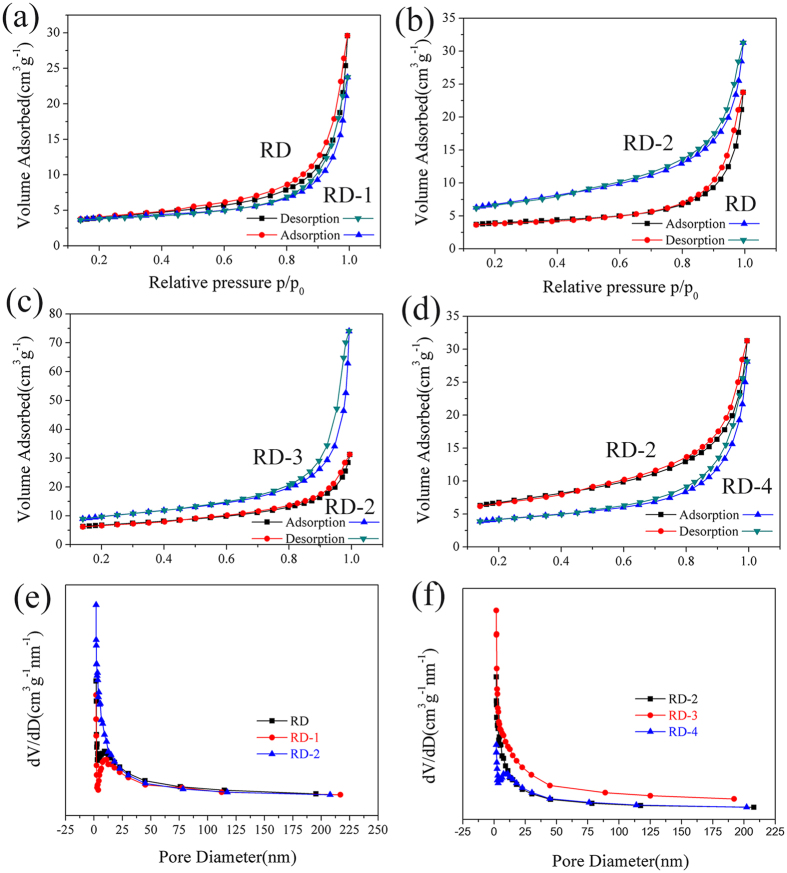
Nitrogen adsorption–desorption isotherms and pore size distributions of raw diatomite and modified samples.

The textural properties of the well-modified diatomite materials, as determined by N_2_ adsorption–desorption isotherms, were preserved after different modification approaches. Figure 3 illustrates the N_2_ adsorption–desorption isotherms of raw diatomite and the modified samples. Table 2 summarizes the textural properties of diatomite modified with different methods. As seen from these figures, the N_2_ adsorption–desorption isotherms of all the samples are similar, indicating that the microstructure of the diatomite is maintained upon modification. Similarly, all the modified samples exhibit adsorption isotherms type H3 hysteresis loop in the relative pressure (p/p_0_) range of 0.4~1.0. The type H3 hysteresis loop, which does not have any adsorption plateau at high relative pressure close to unity, is an indication of the presence of macropores in the materials. However, the relative pressure (p/p_0_) range of 0.4~0.8 is caused by capillary condensation of mesoporous adsorption^28^,^29^. Such a feature suggests the co-presence of macropores and mesopores structures, as shown in SEM and TEM images. From Table 2, after dissolution in alkali solution, three parameters are improved obviously because after the treatment with sodium hydroxide solution, diatomite’s pore type has been changed: large pore size is increased and micropore size is reduced, thus resulting in the increase in the BET surface area. Pore size distribution (PSD) is an important factor for diatomite to be the PCM carrier. Concerning the pore size distribution shown in Fig. 3(e,f), the pore size distribution of alkali-leaching shows a narrower range of 5~10 nm than that of other diatomite samples. It should be noted that the typical pore size distribution of nano-silica decorated diatomite was comparable to the acid-leaching diatomite. For both samples, the pore diameter shifted to larger diameter, which is desirable for PCM carrier.

### Analysis of the prepared PEG/diatomite ss-PCMs

Among these prepared PEG/diatomite ss-PCMs, ss-PCM4, a maximum weight percentage of PEG as high as 70% without any leakage up to a temperature as high as 80 °C, far above the melting temperature of PEG, has been achieved. Such a weight percentage of PEG is the highest value among diatomite-supported shape-stabilized PCMs to the best of our knowledge. Besides, the maximum absorption ratio of PEG is 48%, 51%, 54% and 66%, respectively, by mass percentage of PEG in the ss-PCM1, ss-PCM2, ss-PCM3, ss-PCM5. Fig. S3 gives the detailed exudation stability tests of the prepared PEG/diatomite ss-PCMs in the *Supporting Information.* The results indicated that PEG/diatomite composites prepared in this study is quite stable above the melting temperature.

**Figure 4:**
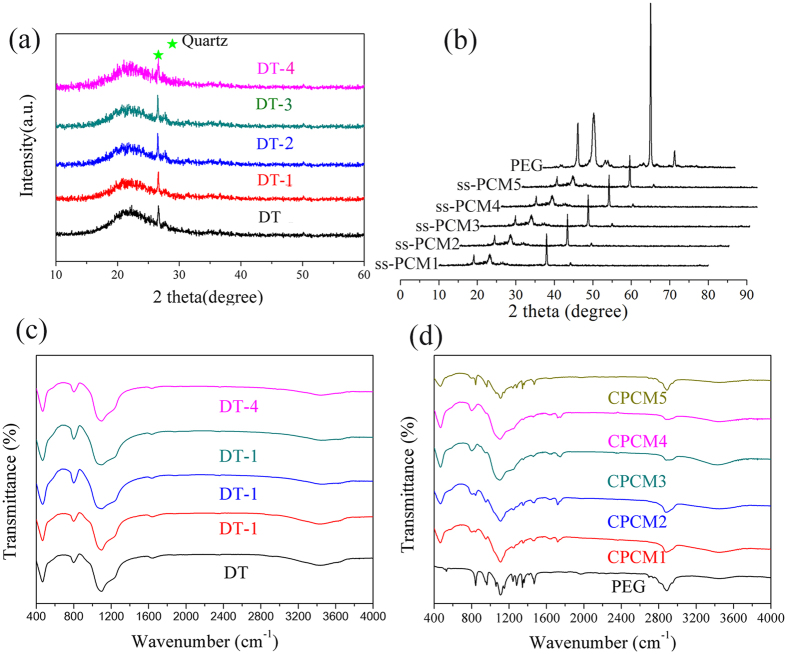
XRD pattern and FTIR spectra of diatomite, PEG and the prepared ss-PCMs.

Chemical compatibility between PEG and diatomite was determined via XRD and FT-IR analysis. The X-ray analysis of the raw diatomite powder before and after modification is given in Fig. 4(a). It can be observed that all the samples have the typical non-crystalline diffraction peaks of SiO_2_. A significant amount of quartz crystalline phase is also found in all samples. Figure 4(b) shows the XRD pattern of PEG and the prepared PEG/diatomite ss-PCMs. In Fig. 4(b), the peaks at 19°, 23°, 38° and 43° are assigned to PEG crystal. It can be clearly seen that all the sharp and intense diffraction peaks of PEG were observed for all the prepared PEG/diatomite ss-PCMs, indicating that the crystal structure of PEG is not destroyed after impregnation.

Figure 4(c,d) demonstrate the FT-IR spectra of modified diatomite, PEG, and ss-PCMs. From the spectrums of in Fig. 4(c), peaks at 1095 and 800 cm^−1^ were respectively caused by Si–O–Si asymmetry stretching vibration and the Si–O–Si symmetric stretching vibration peak. The peak at 466 cm^−1^ can be assigned to the Si–O–Si bending vibration peak. In addition, the stretching vibration and the bending vibration of OH functional group were found at the wave number of 3446 and 1637 cm^−1^, respectively. All of these characteristic peaks suggest that diatomite is mainly composed of SiO_2_. In Fig. 4(d), the triplet peak of the C–O–C stretching vibration at 1143 cm^−1^, 1107 cm^−1^ and 1097 cm^−1^ with a maximum at 1107 cm^−1^ could be clearly observed. Besides, the peaks at 2888 cm^−1^, 962 cm^−1^ and 842 cm^−1^ are caused by the strenching vibration of the –CH_2_ functional group, the crystal peak of PEG and C–C–O bonds. Single –OH functional group was also found at the wave number of 3442 cm^−1^. It is noted that these typical peaks of PEG were all presented in the spectrum of composite PCMs, There was no significant new peak appearing in the spectrum of composite PCMs, which proved that there is no chemical interaction between PEG and diatomite.

**Figure 5:**
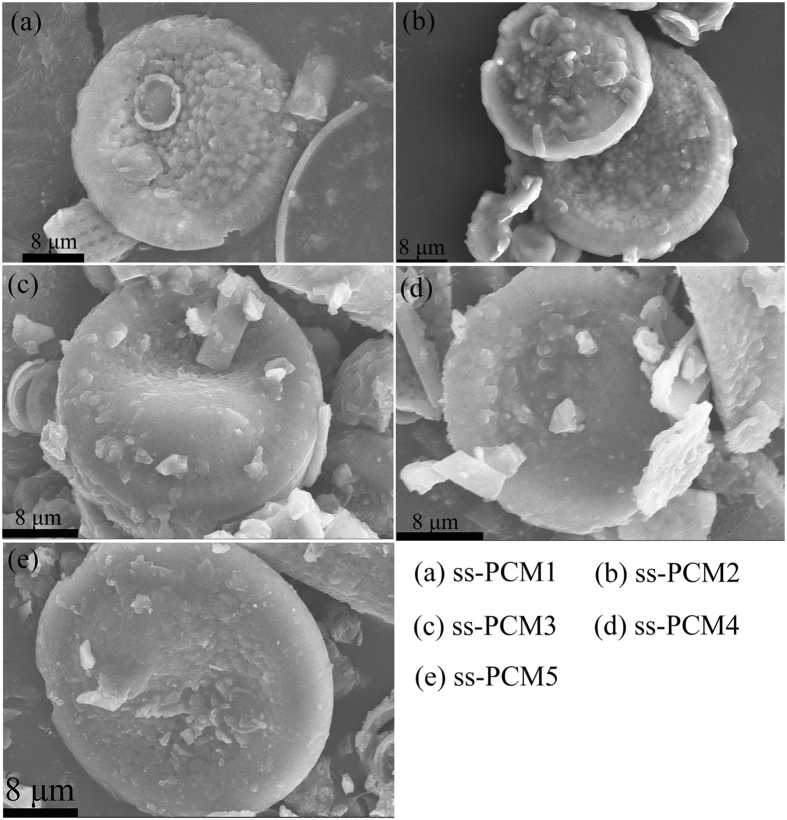
SEM images of the prepared PEG/diatomite ss-PCMs.

Figure 5 demonstrates the microstructures of the prepared PEG/diatomite ss-PCMs. In Fig. 5, the primary porous structure morphology of diatomite as well as interface between PEG and diatomite were not seen. PEG was uniformly absorbed in diatomite with a good physical compatibility. The structure of diatomite provided the good mechanical strength for the whole composite and prevented the seepage of melted PEG due to the effect of capillary and surface tension force. Hence, the shape-stabilized composite PCMs were obtained.

### Phase change behavior of the prepared PEG/diatomite ss-PCMs

**Figure 6:**
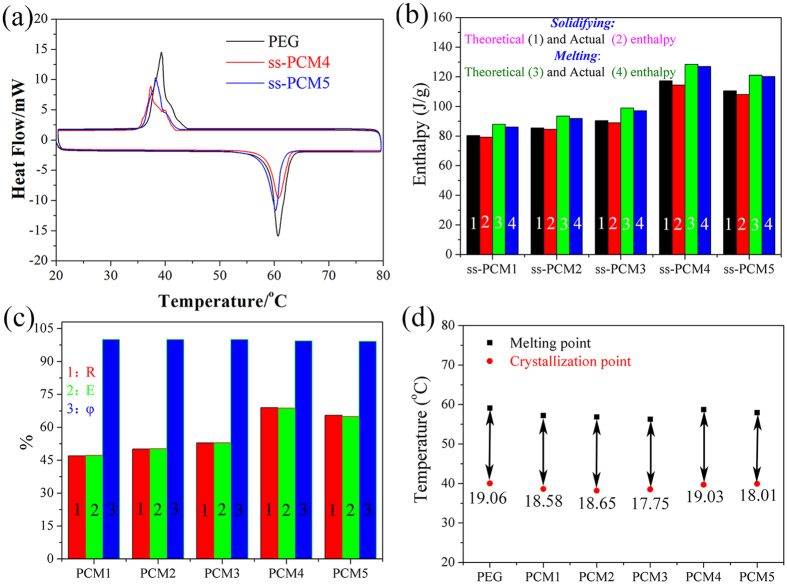
PEG and PEG/diatomite ss-PCMs: (**a**) DSC curves; (**b**) comparison of theoretical and actual enthalpies; (**c**) thermal characteristics; (**d**) phase change temperatures.

Usually, phase changing behavior can be described into thermal energy storage capacity and phase changing temperature, which can be determined by means of DSC technique. Table 3 gives a summary of the characteristic phase change temperatures and the enthalpies. As a critical factor of PCMs, the phase change enthalpy is always considered as a most reliable indicator evaluating the thermal energy storage capacity of the prepared ss-PCMs. In the composites, only the PEG substrate can absorb/release heat during the melting and solidifying process. Therefore, the higher PEG content will positively boost the latent heat storage capacity of the prepared ss-PCM. According to Table 3, ss-PCM4 and ss-PCM5 can be unquestionably considered as the most promising latent heat storage material. Figure 6(a) demonstrates the melting and solidifying DSC curve of ss-PCM4 composite. It is found from Fig. 6(a) that the optimal PEG/diatomite ss-PCM4 melts at 58.75 °C with a latent heat of 127.0J/g and freezes at 39.72 °C with a latent heat of 114.5 J/g when the adsorption ratio of the PEG is 70%. The theoretical enthalpy of ss-PCM could be determined by Eq. (1)^30^:


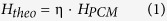


where *H*_*theo*_ represents the theoretical enthalpy of prepared the ss-PCM; η is the mass fraction of the PEG; *H*_*PCM*_ is the actual enthalpy of PEG. However, from Fig.6(b), it is found that the actual enthalpies of ss-PCMs were slightly lower than their theoretical values. The difference may be interpreted as follows: the drag and steric effect of nano- and mesopores restricted the crystal arrangement and orientation of PEG molecular chains, resulting in the decline of regularities of crystal line regions and the increase of lattice defects. Similar results have been obtained by Feng *et al*.^31^. Besides, the melting enthalpy is larger than the solidifying enthalpy. This may be caused by the fact that the mass loss increases when the composite is heated from 25 to 80 °C during melting process of the DSC test. Thereafter, the solidifying process test is carried out. It was due to the mass loss of PEG/diatomite in the melting process that the solidifying enthalpy is smaller in the solidifying process test.

Three significant parameters including the impregnation ratio (R), impregnation efficiency (E), and thermal storage capability (φ) were employed to quantify the phase change performance of the prepared PEG/diatomite ss-PCMs^32^, which can be calculated using the following three equations:


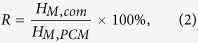



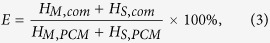



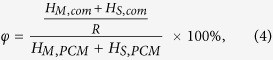


where *H*_*M,com*_ and *H*_*S,com*_ represent the melting and solidifying phase change enthalpies, respectively; *H*_*M, PCM*_ and *H*_*S, PCM*_ respectively denote the melting latent heat and solidifying latent heat of PEG. R represents the effective impregnation of PEG within the structure of diatomite, while E describes an effective performance of the PEG inside the composite for latent heat storage. The calculated results of R, E, and φ of all the prepared ss-PCMs samples are shown in Fig. 6(c). Importantly, the thermal storage capability of all the prepared ss-PCMs samples is chose to 100%, indicating that almost all PEG molecule chains could effectively store/release heat through phase transition. In detail, the prepared ss-PCM4 achieved a highest impregnation ratio of 69.2% and efficiency of 68.8%, when prepared at the PEG and DtAg mass ratio of 70/30. The two parameters are lower than the theoretical PEG mass ratio. The phase change can hardly occur on the PEG inside the tiny pores due to the confinement effect on molecule motion, that is, not all the PEG in ss-PCM4 could actually serve as PCMs.

The extent of supercoiling could be calculated as the difference between the melting and solidifying temperature. The corresponding evaluation results are described in Fig. 6(d). Compared with the pure PEG PCM, the extent of supercooling in the prepared PEG/diatomite ss-PCMs is reduced by 2~7%. This suggests that the extent of supercooling of PEG can be favorably reduced by impregnation with diatomite.

### NEvaluation of the activation energy

**Figure 7:**
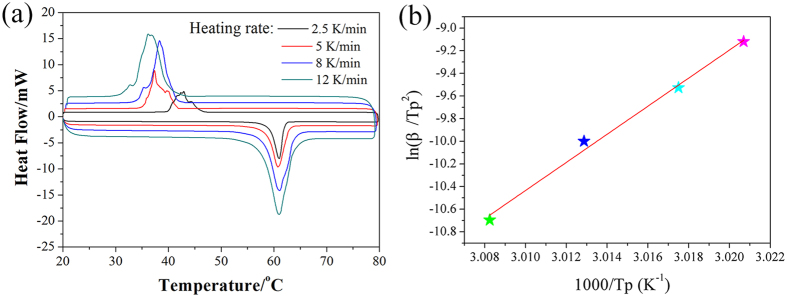
(**a**) DSC curves of ss-PCM4 at different heating rate; (**b**) Kissinger plot of the ss-PCM4.

Interestingly, if the heating rate is changed, the peak temperature and latent heat on the DSC curve are also changed. Figure 7(a) shows the DSC curves of ss-PCM4 tested with different heating rates (2.5~12 K/min). And the corresponding thermodynamic parameters calculated are summarized in Table 4. As shown in Fig. 7(a), no two phase region emerges for and the shape of the thermogram is similar to that for pure PEG. Besides, it is noted that the melting temperature range becomes broader and it shifts to lower temperature with increasing heating rate. Based on Table 4, the enthalpies decline along with the heating rate increment.

According to the literature^33^, the variation in the peak temperature with heating rate is governed only by the activation energy (*Ea*), which can be determined in terms of the Kissinger equation (5):


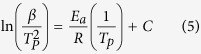


where *β* is the heating rate; *Tp* denotes the maximum melting endothermic peak; *Ea* represents the apparent activation energy of the PEG^34^,^35^. Plotting *ln (β/T*_*P*_^2^) versus *1/T*_*P*_ should give a straight line. *Ea/R*, as the slope of the line, is employed to determine the apparent activation energy. Figure 7(b) illustrates the Kissinger plot for the ss-PCM4. After calculation, the apparent activation energy for PEG in the ss-PCM4 was determined to be 1031.85 kJ·mol^−1^, which is higher than that of pure pristine PEG.

### Thermal stability of the prepared PEG/diatomite ss-PCMs

**Figure 8:**
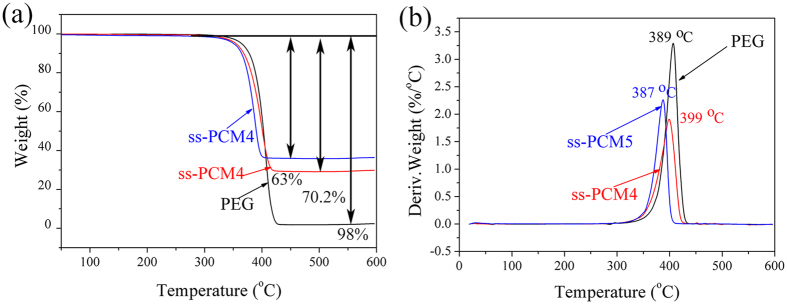
TGA curves and the corresponding DTG thermograms of pristine PEG and PEG/diatomite ss-PCMs.

Thermal stability is a critical parameter for ss-PCMs. Figure 8 displays TGA curves and DTG thermograms of PEG, ss-PCM4 and ss-PCM5. Table 5 lists the obtained characteristic temperatures and residual masses. As shown in Fig. 8, PEG exhibits a typical one-step weight loss at 389 °C, suggesting that the pristine PEG experienced a simple evaporation. TGA curves of the prepared ss-PCMs were almost overlapped with that of PEG at the low temperature zone, showing that the composites have good thermal stability before 350 °C. Corresponding to the degradation process, the sharp weight loss within the temperature around 400 °C can be ascribed to the decomposition of organic ingredients, namely the breaking of the PEG chains. From Table 5, only 2 wt% of residue was obtained at 600 °C for PEG. The composites had higher residue of 29.8 wt% and 37 wt% at 600 °C, suggesting that the prepared PEG/diatomite ss-PCMs were homogeneous.

### Durability test of the prepared ss-PCM4

**Figure 9:**
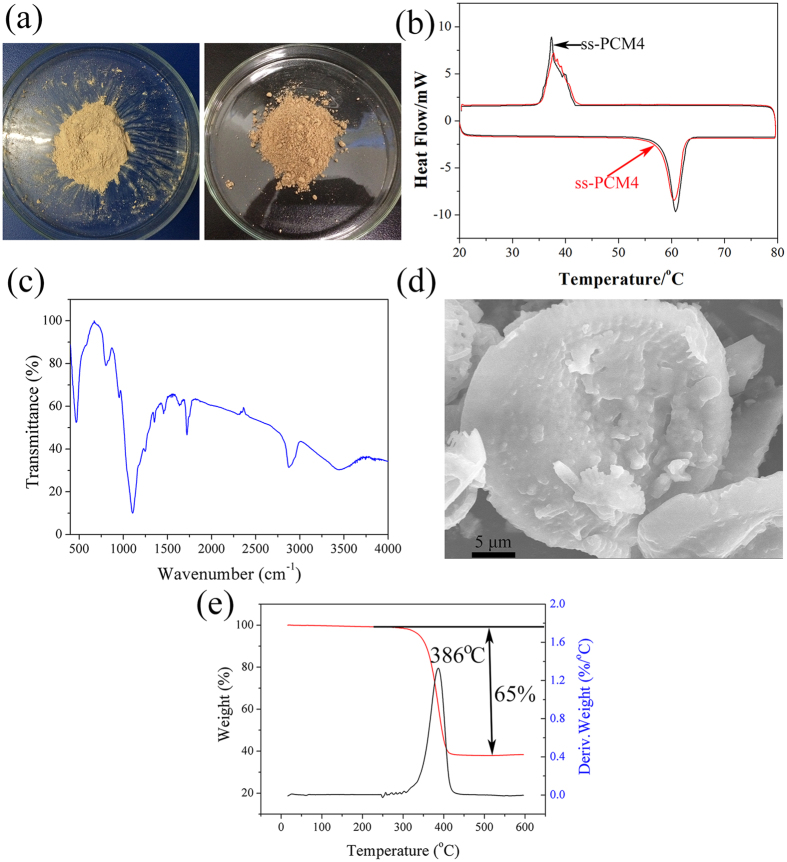
The ss-PCM4 after 200 cycling: (**a**) photographs; (**b**) DSC curves; (**c**) FTIR spectra; (**d**) SEM image; (**e**) TGA curve and the corresponding DTG thermogram.

The composite PCM should be stable in terms of thermal and chemical manner over a large number of melting and freezing cycling. That is, there should be no or less change in its thermal properties and chemical structure after long-term utility period. Thus, 200-cycle test was performed on the ss-PCM4 to determine its long-term stability. Figure 9(a) shows the macroscopic photographs of ss-PCM4 before and after the thermal cycle experiment. No obvious changes were observed in physical appearance. Figure 9(b) shows the DSC curves of the ss-PCM4 before and after the thermal cycle test. After 200 thermal cycles, the melting temperature of composite PCM was changed by 0.65 °C. The solidifying temperature was changed by 0.41 °C. Besides, the melting enthalpy was changed by 7.1% and solidifying enthalpy was changed by 4.8%. This was probably caused by the leakage of the PEG attached on the surface or within the pores due to the thermal expansion during the melting.

Moreover, it is substantiated by comparing the chemical and morphology characterization of ss-PCM4 before and after 200 thermal cycle experiment with FT-IR and SEM techniques. As seen from the spectra in Fig. 9(c), the shape and frequency values of all the peaks did not change after the thermal cycling. The result indicated that the chemical structure of the composite PCM was not affected by the repeated melting and freezing cycling. From Fig. 9(d), it is clear seen that the morphology of disc structure is intact. PEG is fully dispersed into diatomite pores and no empty pores are found.

On the other hand, the TGA curve and DTG thermogram of the composite PCM after thermal cycling are shown in Fig. 9(e). As can be seen from the curves, the degradation of PEG started at 360 °C. The result showed that the composite PCM has good thermal stability even after the thermal cycling. Note that there was a 35 wt% residual remained, which is well consistent with the corresponding DSC analysis. In conclusion, the phase change properties of the composite PCM were maintained after 200 thermal cycles, without loss of their morphologies and stability.

### Vacuum immersion results

**Figure 10:**
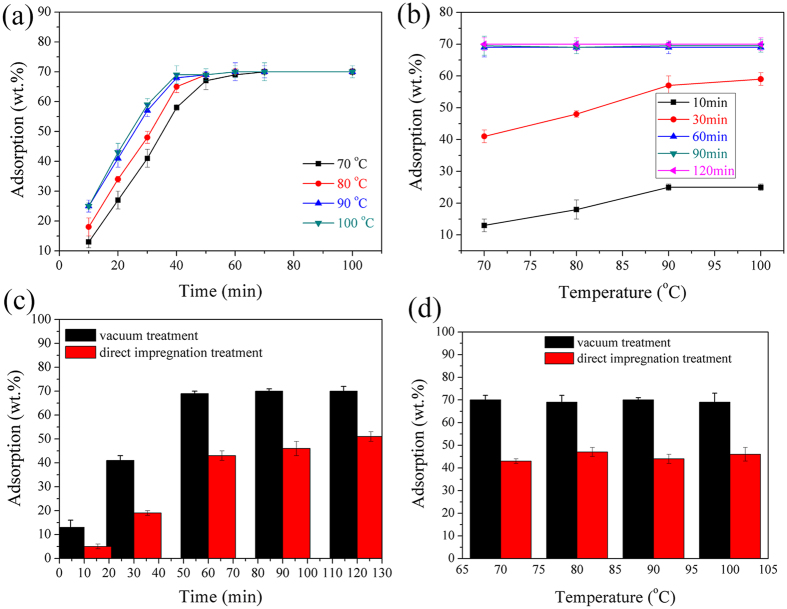
(**a**) and (**b**): Effects of immersion time and temperature on PEG adsorption capacity; (**c**) and (**d**): Comparison between vacuum and direct impregnation method.

The effects of immersion time and temperature on the PEG adsorption capacity are shown in Figure 10(a,b). From Fig. 10(a), in the initial 60 min, the adsorption capacity of diatomite increases rapidly. But after 60 min, the value remains almost unchanged. From Fig. 10(b), in the 10-min and 30-min immersion, the adsorption capacity increases when the temperature rises to 90 °C and remains nearly unchanged. After 60 min, the immersion temperature has a slight effect on the adsorption capacity. Therefore, the immersion time of 60 min and the immersion temperature of 90 °C were regarded as the optimal conditions for the preparation of PEG/diatomite ss-PCMs.

**Figure 11:**
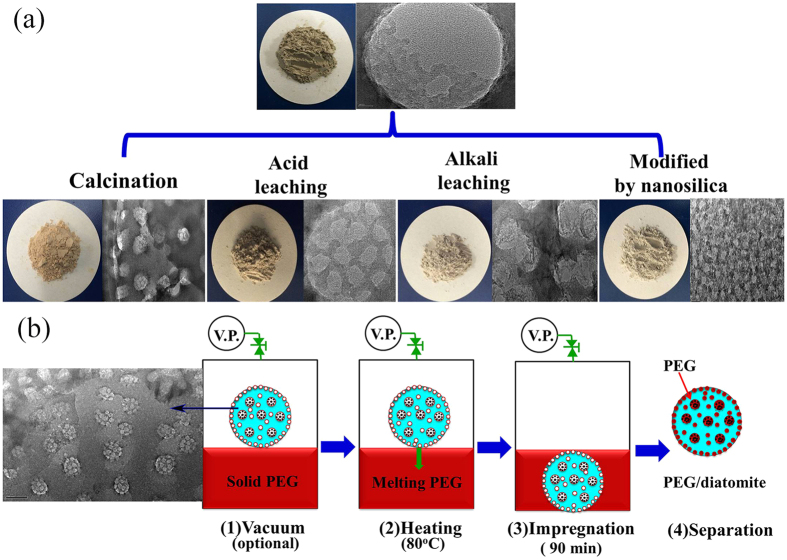
(**a**) Schematic routes for the diatomite modification; (**b**) Image of vacuum impregnation treatment for preparing phase change composites (VP: vacuum pump).

According to the literatures^5^,^18^,^19^,^20^, little difference was observed in the composite PCMs prepared with the natural immersion in air. Thus, the two methods were compared and the results are shown in Fig. 11(c,d). Based on the two figures, it is quite clear that the composite PCM prepared using the vacuum treatment own higher PEG ratio than that with natural immersion. Because of the unique network porosity and high surface affinity of diatomite, PEG was well impregnated and trapped into the pores of diatomite due to the capillary forces. But the atmosphere pressure within the pores prevents the immersion. Usually, it is more difficult for liquid PEG to permeate into the pore space with smaller diameter than that with larger diameter. Thus, for a supporting substrate with hierarchical pores, vacuum immersion method is more effective and required. Moreover, those composite PCMs, whose pores are not full of PEG, could not be applied because air within the pores may expand dramatically at the elevated temperature and cause the PEG leakage.

### Comparison with other studies

The comparative studies of thermal properties of the prepared PEG/diatomite ss-PCMs with that of diatomite-supported PCMs in literature^5^,^6^,^17^,^18^,^19^,^20^,^36^,^37^ are summarized in Table 6. From Table 6, the prepared ss-PCM4 has the highest impregnation ratio of PEG as high as 70 wt% and the largest phase change enthalpies. In addition, the prepared ss-PCM5 has the second PEG impregnation ratio of 66 wt%. To the best of our knowledge, Nomura *et al*.^20^ reported that the maximum load of PEG in diatomite powder could reach 61 wt% while PEG melts during solid-liquid phase transformation. As known, in the PCM composites, only the PEG substrate can absorb/release heat during the melting and solidifying process. Therefore, the higher PEG content will positively boost the latent heat storage capacity of the prepared ss-PCMs, which is quite desirable for practical application. Compared with other phase change materials, the composite PEG ss-PCMs in this study using alkali-leaching and nano-silica decorated diatomite as supporting substrate have better form-stable ability, larger enthalpy, and a more extensive future for further modification. Thus, they were noticeably suitable for thermal energy storage applications in the exterior envelopes of buildings.

## Discussion

A series of novel diatomite-supported shape-stabilized composite PCMs were prepared via a vacuum impregnation operation. The modification effects of the four methods including calcination, acid treatment, alkali leaching and nano-silica decoration on diatomite were studied. Based on the above discussion, the conclusions can be drawn as follows:

Composite PCMs were prepared at 90 °C for 60 min. The maximum load of PEG in the composite ss-PCM4 could reach 70 wt% using the alkali-leaching diatomite, which was 46% higher than that of the raw diatomite. The maximum load of PEG in the composite ss-PCM5 could reach 66 wt% using the nano-silica decorated diatomite. 8 min alkali treatment (5%, 70 °C) could dredge and enlarge the pores leading to higher BET area, higher pore size. After decorated by nano-silica, the multi-pore structure greatly contributes to higher BET area. These methods were proved to be facile and effective for PEG adsorption.

The resulting PEG/diatomite ss-PCMs own high exudation stability even in the liquid state of PEG. SEM results indicated that PEG was well impregnated. FT-IR, XRD, DSC, TGA and 200-cycle experiment results proved the excellent chemical compatibility, improved supercooling extent, stability and reliability.

The resulting 70%PEG/diatomite composite has suitable phase change temperature and largest enthalpies with the apparent activation energy of 1031.85 kJ· mol^−1^, which was nearly twice higher than pristine PEG PCM. It could be unquestionably chosen as the most promising latent heat storage material for applications in building envelopes in the continuous hot summer.

## Methods

The raw diatomite samples used in the experiments were obtained from Changbai Mountain, Ltd. Before the composite PCMs preparation, the raw diatomite (denoted as RD) was well modified to improve its PEG adsorption capacity. Figure 11(a) gives the schematic routes for the RD sample modification. As shown in Fig. 11(a), firstly, RD particles were thermally treated at 400 °C for 4 h, which was denoted as RD-1. The obtained RD-1 powder was secondly processed by acid-leaching in 20 wt% H_2_SO_4_ solution at 80 °C for 2 h, which was denoted as RD-2. Thirdly, the resulting RD-2 powder was immersed in sufficient amount of 5 wt% sodium hydroxide solution at 70 °C for 8 min to obtain RD-3. At last, RD-2 powder was also decorated with nano-silica through a similar facile temperature-assisted sol–gel method^7^. The final product was denoted as RD-4. All the detailed laboratory procedures and the experimental matters needing attention were given in the *Supporting Information.*

The PEG (Mw = 4000) PCM was supplied from Beijing Chemical Reagent Ltd. The PEG latent heat was 183.4 J/g at the melting temperature of 59.13 °C and 167.6 J/g at the freezing temperature of 40.07 °C. Figure 11(b) illustrates the procedure of impregnation treatment with/without vacuuming. The detailed operation procedures were also given in the *Supporting Information*. During the vacuum treatment, the impregnation time in liquid PCM was changed from 10 to 120 min to study the effect of time on adsorption capacity. Besides, the impregnation temperature was controlled at 70~120 °C to investigate the effect of temperature on adsorption capacity. Moreover, the results of adsorption capacity obtained with/without vacuuming were compared. The resulting PEG/diatomite ss-PCMs were respectively denoted as ss-PCM1, ss-PCM2, ss-PCM3, ss-PCM4, ss-PCM5 based on RD, RD-1, RD-2, RD-3, RD-4 support. Further characterizations technologies of the modified diatomite and the prepared composite PCMs were summarized in the *Supporting Information.*

## Additional Information

**How to cite this article**: Qian, T. *et al*. Pore structure modified diatomite-supported PEG composites for thermal energy storage. *Sci. Rep.* **6**, 32392; doi: 10.1038/srep32392 (2016).

## Supplementary Material

Supplementary Information

## Figures and Tables

**Table 1:** Chemical compositions of diatomite before and after modification.

Samples	Ingredients (wt%)									
	SiO_2_	Al_2_O_3_	TFe_2_O_3_	MgO	CaO	Na_2_O	K_2_O	TiO_2_	P_2_O_5_	MnO
RD	86.82	3.21	1.60	0.43	0.46	0.25	0.56	0.15	0.22	0.011
RD-1	92.78	3.44	1.68	0.46	0.46	0.27	0.60	0.16	0.23	0.011
RD-2	93.18	1.68	0.23	0.13	0.14	0.32	0.32	0.09	0.17	0.004
RD-3	87.63	1.42	0.48	0.88	1.18	0.53	0.45	0.16	0.10	0.005
RD-4	94.43	1.64	0.23	0.13	0.14	0.31	0.30	0.09	0.18	0.005

**Table 2:** Textural properties of raw diatomite and modified samples.

Results	Samples				
	RD	RD-1	RD-2	RD-3	RD-4
Specific surface area (m^2^/g)	13.8021	14.195	24.0548	35.0635	29.8231
Pore volume (cc/g)	0.0241	0.0290	0.0362	0.0718	0.04277
Mean Pore Diameter (nm)	6.02	6.99	8.18	9.36	7.97

**Table 3:** Thermal characteristics of PEG and the prepared PEG/diatomite ss-PCMs.

Samples	PEG Mass Ratio (wt%)	Melting Process		Solidifying Process	
		H_M_(J/g)	T_M_(°C)	H_S_(J/g)	T_S_(°C)
PEG	100	183.4	59.13	167.6	40.07
ss-PCM1	48	86.2	57.21	79.3	38.63
ss-PCM2	51	91.9	56.86	84.6	38.21
ss-PCM3	54	97.1	56.29	89.0	38.54
ss-PCM4	70	127.0	58.75	114.5	39.72
ss-PCM5	66	120.2	57.96	108.1	39.95

**Table 4:** Thermal characteristics of PEG/diatomite at different heating/cooling rate.

Sample	Heating/cooling rate (K/min)	Melting Process		Solidifying Process	
		H_M_(J/g)	T_M_(°C)	H_S_(J/g)	T_S_(°C)
ss-PCM4	2.5	130.1	59.26	118.6	42.93
ss-PCM4	5.0	127.0	58.75	114.5	39.72
ss-PCM4	8.0	128.9	58.24	116.1	38.33
ss-PCM4	12.0	123.7	57.89	110.1	36.16

**Table 5:** TGA data of pristine PEG and the prepared PEG/diatomite ss-PCMs.

Samples	Onset (°C)	Peak (°C)	Endset (°C)	Residual mass(%)
PEG	389	407	417	2.0
ss-PCM4	376	399	411	29.8
ss-PCM5	370	387	396	37.0

**Table 6:** Comparison of the thermal properties of the prepared ss-PCM with that of PEG/diatomite in literature.

PCM	Diatomite Pretreatment	Method	Maximum Ratio	Melting Process		Solidifying Process		Ref.
				H_m_(J/g)	T_M_(°C)	Hs(J/g)	T_S_(°C)	
Paraffin	Calcination	Fusion adsorption	61.0%	89.54	33.04	89.80	52.43	[14]
PEG	—	Vacuum impregnation	50.0%	87.09	27.70	82.22	32.19	[6]
Paraffin	—	Fusion adsorption	50.0%	63.98	22.30	—	—	[19]
Paraffin	Calcination (600 °C, 2 h)	Direct impregnation	47.4%	70.51	47.81	71.96	44.21	[18]
Fatty acid	—	Fusion adsorption	40.0%	117.12	16.36	—	—	[17]
PEG	Calcination & Acid leaching	Direct impregnation	55.0%	103.70	58	92.08	40	[5]
Na_2_SO_4_	—	Fusion adsorption	55.0%	73.9	882	—	—	[36]
n-hexadecane n-octadecane Paraffin	—	Vacuum impregnation	50.0%	120.1 116.8 61.96	23.68 31.29 54.24	118.0 112.9 59.74	13.17 23.65 50.23	[37]
PEG	NaOH Leaching	Vacuum impregnation	70%	127.0	58.75	114.5	39.72	present paper
PEG	Nano-silica Decoration	Vacuum impregnation	66%	120.2	57.96	108.1	39.95	present paper

## References

[b1] Crabtree G. W. & Lewis N. S. Solar energy conversion. Phys. Today 60, 37–42 (2007).

[b2] Zeng J. L. . Preparation and thermal properties of palmitic acid/polyaniline/exfoliated graphite nanoplatelets shape-stabilized phase change materials. Appl. Energy 115, 603–609 (2014).

[b3] Zhong L. M. . Preparation and thermal properties of porous heterogeneous composite phase change materials based on molten salts/expanded graphite. Sol. Energy 107, 298–306 (2014).

[b4] Li W. H., Song G. L., Li S. H., Yao Y. W. & Tang G. Y. Preparation and characterization of novel MicroPCMs (microencapsulated phase-change materials) with hybrid shells via the polymerization of two alkoxy silanes. Energy 70, 63–73 (2014).

[b5] Qian T. T., Li J. H., Ma H. W. & Yang J. Adjustable thermal property of polyethylene glycol/diatomite shape-stabilized composite phase change material. Polym. Compos. 37, 854–860 (2016).

[b6] Karaman S., Karaipekli A., Sarı A. & Biçer A. Polyethylene glycol (PEG) /diatomite composite as a novel shape-stabilized phase change material for thermal energy storage. Sol. Energy Mater. Sol. Cells 95, 1647–1653 (2011).

[b7] Qian T. T., Li J. H. Ma H. W. & Yang J. The preparation of a green shape-stabilized composite phase change material of polyethylene glycol/SiO_2_ with enhanced thermal performance based on oil shale ash via temperature-assisted sol–gel method. Sol. Energy Mater. Sol. Cells 132, 29–39 (2015).

[b8] Min X. . Enhanced thermal properties of novel shape-stabilized PEG composite phase change materials with radial mesoporous silica sphere for thermal energy storage. Sci. Rep. 5 (2015). 10.1038/srep12964 PMC4531330 26261089

[b9] Zhang D., Zhou J., Wu K. & Li Z. J. Granular phase change composites for thermal energy storage. Sol. Energy 78, 351–480 (2005).

[b10] Yu S., Wang X. & Wu D. Microencapsulation of n-octadecane phase change material with calcium carbonate shell for enhancement of thermal conductivity and serving durability: synthesis, microstructure, and performance evaluation. Appl. Energy 114, 632–643 (2014).

[b11] Li M., Wu Z., Kao H. & Tan J. Experimental investigation of preparation and thermal performances of paraffin/bentonite composite phase change material. Energy Convers. Manage. 52, 3275–3281 (2011).

[b12] Ling T. C. & Poon C. S. Use of phase change materials for thermal energy storage in concrete: an overview. Constr. Build. Mater. 46, 55–62 (2013).

[b13] Al-Degs Y., Khraisheh M. A. M. & Tutunji M. F. Sorption of lead ions on diatomite and manganese oxides modified diatomite. Water Res. 35, 3724–3728 (2001). 11561635 10.1016/s0043-1354(01)00071-9

[b14] Sun Z. M., Zhang Y. Z., Zheng S. L., Park Y. & Frost R. L. Preparation and thermal energy storage properties of paraffin/calcined diatomite composites as form-stable phase change materials. Thermochim. Acta. 558, 16–21 (2013).

[b15] Hadjar H. . Elaboration and characterisation of new mesoporous materials from diatomite and charcoal. Micropor. Mesopor. Mat. 107, 219–226 (2008).

[b16] Garderen N., Clemens F. J., Mezzomo M., Bergmann C. P. & Graule T. Investigation of clay content and sintering temperature on attrition resistance of highly porous diatomite based material. Appl. Clay Sci. 52, 115–121 (2011).

[b17] Li M., Kao H., Wu Z. & Tan J. Study on preparation and thermal property of binary fatty acid and the binary fatty acids/diatomite composite phase change materials. Appl. Energy 88, 1606–1612 (2011).

[b18] Xu B. & Li Z. Paraffin/diatomite composite phase change material incorporated cement-based composite for thermal energy storage. Appl. Energy 105, 229–237 (2013).

[b19] Li X. Y., Sanjayan J. G. & Wilson J. L. Fabrication and stability of form-stable diatomite/paraffin phase change material composites. Energy Build. 76, 284–294 (2014).

[b20] Nomura T., Okinaka N. & Akiyama T. Impregnation of porous material with phase change material for thermal energy storage. Mater. Chem. Phys. 115, 846–850 (2009).

[b21] Sarı A. & Bicer A. Thermal energy storage properties and thermal reliability of some fatty acid esters/building material composites as novel form-stable PCMs. Sol. Energy Mater. Sol. Cells 101, 114–122 (2012).

[b22] Li E., Zeng X. Y. & Fan Y. H. Removal of chromium ion (III) from aqueous solution by manganese oxide and microemulsion modified diatomite. Desalination 238, 158–165 (2009).

[b23] Zhang G. L. . Microstructural modification of diatomite by acid treatment, high-speed shear, and ultrasound. Micropor. Mesopor. Mater. 165, 106–112 (2013).

[b24] Franca S., Millqvist M. & Luz A. Beneficiation of Brazilian diatomite for the filtration application industry. Miner. Metall. Process. 20, 42–47 (2003).

[b25] Jung K. W., Jang D. & Ahn K. H. A novel approach for improvement of purity and porosity in diatomite (diatomaceous earth) by applying an electric field. Int. J. Miner. Process 131, 7–11 (2014).

[b26] Sun Z. M., Yang X. P., Zhang G. X., Zheng S. L. & Frost R. L. A novel method for purification of low grade diatomite powders in centrifugal fields. Int. J. Miner. Process 125, 18–26 (2013).

[b27] Zhang J., Ping Q. W., Niu M. H., Shi H. Q. & Li N. Kinetics and equilibrium studies from the methylene blue adsorption on diatomite treated with sodium hydroxide. Appl. Clay Sci. 83–84, 12–16 (2013).

[b28] Jin J., Ouyang J. & Yang H. M. One-step synthesis of highly ordered Pt/MCM-41 from natural diatomite and the superior capacity in hydrogen storage. Appl. Clay Sci. 99, 246–253 (2014).

[b29] Du Y. C., Yan J., Meng Q., Wang J. S. & Dai H. X. Fabrication and excellent conductive performance of antimony-doped tin oxide-coated diatomite with porous structure. Mater. Chem. Phys. 133, 907–912 (2012).

[b30] Qian Y. . Preparation of a novel PEG composite with halogen-free flame retardant supporting matrix for thermal energy storage application. Appl. Energy 106, 321–327 (2013).

[b31] Feng L. L. . The shape-stabilized phase change materials composed of polyethylene glycol and various mesoporous matrices (AC, BA-15 and MCM-41). Sol. Energy Mater. Sol. Cells 95, 3550–3556 (2011).

[b32] Yu S. Y., Wang X. D. & Wu D. Z. Microencapsulation of n-octadecane phase change material with calcium carbonate shell for enhancement of thermal conductivity and serving durability: synthesis, microstructure, and performance evaluation. Appl. Energy 114, 632–643 (2014).

[b33] Feng L. L. . Preparation and characterization of polyethylene glycol/active carbon composites as shape-stabilized phase change materials. Sol. Energy Mater. Sol. Cells 95, 644–650 (2011).

[b34] Kissinger H. E. Variation of peak temperature with heating rate in differential thermal analysis. J. Res. Nat. Bur. Stan. 57, 217–221 (1956).

[b35] Kissinger H. E. Reaction kinetics in differential thermal analysis. Anal Chem 29, 1702–1706 (1957).

[b36] Qin Y. . Sodium sulfate–diatomite composite materials for high temperature thermal energy storage. Powder Technol. 282, 37–42 (2015).

[b37] Jeong S. G., Jeon J., Lee J. H. & Kim S. Optimal preparation of PCM/diatomite composites for enhancing thermal properties. Int. J. Heat Mass Tran. 62, 711–717 (2012).

