# Supplementary material for: Pore structure modified diatomite-supported PEG composites for thermal energy storage
Source: Sci Rep. 2016 Sep 1;6:32392. doi: 10.1038/srep32392 (PMC5007675; doi:10.1038/srep32392)
Supplement: Supplementary Information [file srep32392-s1.pdf]

Pore structure modified diatomite-supported PEG composites for thermal energy storage

Tingting Qian,* Jinhong Li*, Yong Deng

Beijing Key Laboratory of Materials Utilization of Nonmetallic Minerals and Solid Wastes, National Laboratory of Mineral Materials, School of Materials Science and Technology, China University of Geosciences (Beijing), Beijing 100083, P.R. China

2. Experimental

2.1 Raw materials and modification technique of diatomite carrier

As known, raw diatomite (RD) consists of mainly amorphous silica and several types of organic and inorganic impurities such as ferric oxide, alumina, alkali and alkaline-earth metal oxides, etc [1]. Most of these impurities usually exist in the pores of RD making the pores clogged and thus the pore size reduced, which is unfavorable for the PCM composites preparation. Consequently, dredging the clogged pores and enlarging the pore size are crucial to improve the quality of diatomite as a PCM support.

2.1.1. Calcination

As shown in Fig. 1(a), after calcination, the external color of RD-1 changed from grey to pink, which might be attributed to the valence change of iron because of oxidative reaction.

---

*Corresponding author.

E-mail: jinhong@cugb.edu.cn (Jinhong Li), qiantt2007@cugb.edu.cn (Tingting Qian).

2.1.2. Acid treatment

Acid treatment was used to remove the fine impurities and to have well-opened pores on the porous structure of the diatomite. In a typical acid leaching step, 40 g RD-1 powders and 160 mL 20 wt.% sulfuric acid were added into a 3-mouth flask in the heated thermostatic water bath, which was connected to a mechanical agitator equipped with twin-bladed impeller. The mixture was heated to 80 ℃ (±0.5 ℃) for 2 h. Then, the slurry was filtered and the residue was washed with distilled water for several times until the pH value reached 7. The acid treated diatomite was dried at 105 ℃ for 24 h, ground into to powder, and then kept in desiccators (denoted as RD-2).

2.1.3. Alkali leaching

Diatomite was treated with sodium hydroxide to enhance its performance as a carrier. The RD-2 samples were immersed in sufficient amount of 5% (w/w) sodium hydroxide solution at 70 ℃ for 8 min. The digested diatomite was washed several times by deionized water, filtered, dried at 105 ℃, sieved and stored in closed containers for further tests, which was denoted as RD-3.

2.1.4. Decorate by nano-silica particles

The temperature-assisted sol–gel method was employed to prepare the nano-SiO_2 decorated diatomite [2]. In a typical experimental operation, 5 g RD-2 powder was dissolved in 50 mL of Na_2SiO_3 solution (0.03 mol L^{-1}) under stirring for overnight and the temperature was controlled at 60 ^oC in a constant temperature bath. Then

acetic acid solution (10 wt.%) was gradually added into the solution under continuous stirring to form silica gel until the pH value reached to 4. After aging for 2 h, the composite powder was washed with distilled water for three times and then collected. The products were dried in a vacuum oven at 80 ^{o}C for 48 h. Finally, the nano-SiO_2 decorated diatomite was obtained and denoted as RD-4.

2.2 Preparation of PEG/diatomite ss-PCMs

20 g of diatomite sample was placed inside a filtering flask, which was connected to a vacuum pump apparatus to evacuate air from its porous surface. Then, the valve between the flask and a container filled with liquid PEG was opened to let liquid PEG flow into the flask to cover the diatomite sample. After a period of time, air was allowed to enter the flask again to force the liquid PEG to penetrate into the pore space of diatomite. The porous diatomite materials filled with the PEG were taken out from liquid PCM and then for removing liquid PCM captured by the surface of composites or not supported in pore. They were kept in the furnace which was keeping at 80 ℃. Finally, the products were taken out from the furnace and dried.

2.3 Analysis methods

The specific surface area and pore volume of diatomite were determined by a N_2 adsorption analyzer (Quantachrome Instruments, US). Transmission electron microscope (TEM, JEM-2100HR, Japan) and scanning electronic microscope (SEM, Model HITACHI S-4800) was adopted to observe the microstructures of the modified diatomite and the prepared ss-PCMs. The chemical compatibility of ss-PCMs was

obtained via Fourier transform infrared spectroscopy (FT-IR, Model Frontier) and X-ray diffraction (XRD, Model XD-3) method. Besides, thermal property and stability of the ss-PCMs were explored through differential scanning calorimeter (DSC, Q2000) and thermo-gravimetric analysis (TGA, Q50), respectively.

3 Results and discussion

3.1 Characterization of modified diatomite

3.1.1. Morphology observation

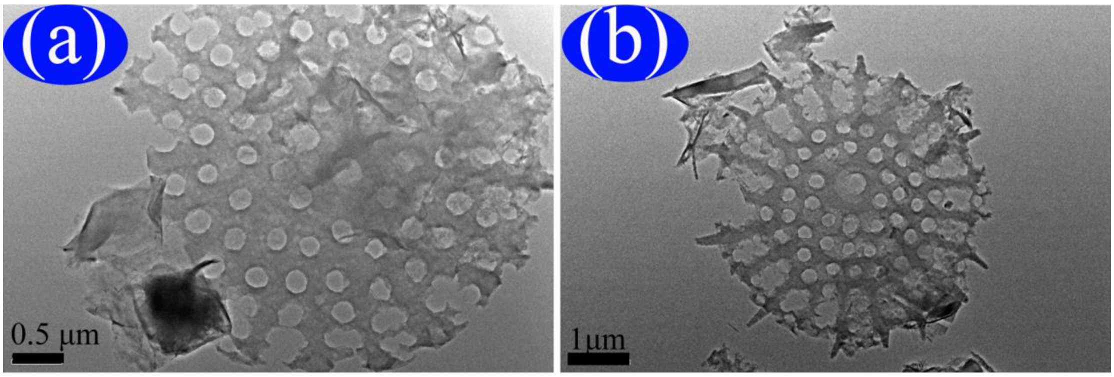

Fig. S1 TEM images of diatomite treated with 5% NaOH with different time: (a) 12 min; (b) 18 min

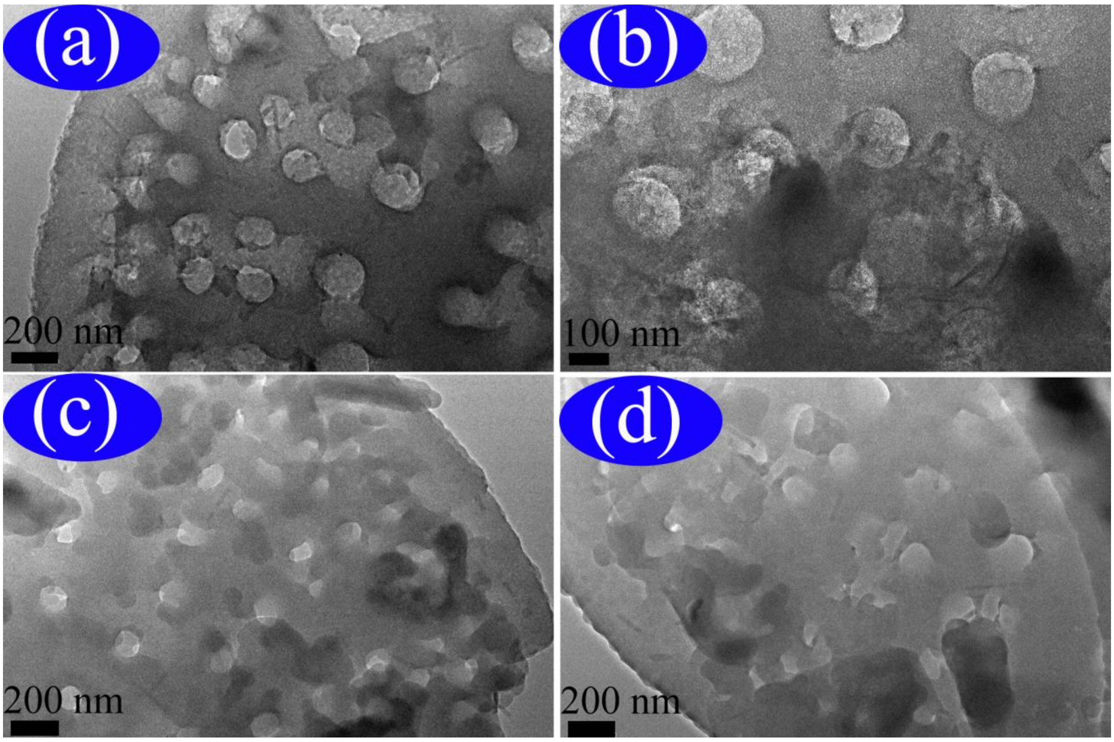

Fig. S2 TEM images of diatomite decorated with nano-silica particles using different Na_2SiO_3 initial concentrations: (a)0.04 mol L^{-1} ; (b) 0.05 mol L^{-1}; (c) 0.08 mol L^{-1}; (d) 0.1 mol L^{-1}

3.2. Analysis of the prepared PEG/diatomite ss-PCMs

3.2.1. Exudation stability of the prepared PEG/diatomite ss-PCMs

The prepared PEG/diatomite ss-PCMs were heated to 80 ^{o}C for 2–10 h to investigate their exudation stability. The macroscopic photographs of different modified diatomite, ss-PCMs, and pristine PEG after heating are presented in Fig. S3(a). No liquid PEG was observed on the surface of PEG/diatomite composite. In addition, from Fig. S3(b), the mass loss during the melting process can be neglected. Based on the results of the above two methods, PEG/diatomite composite prepared in this study is quite stable.

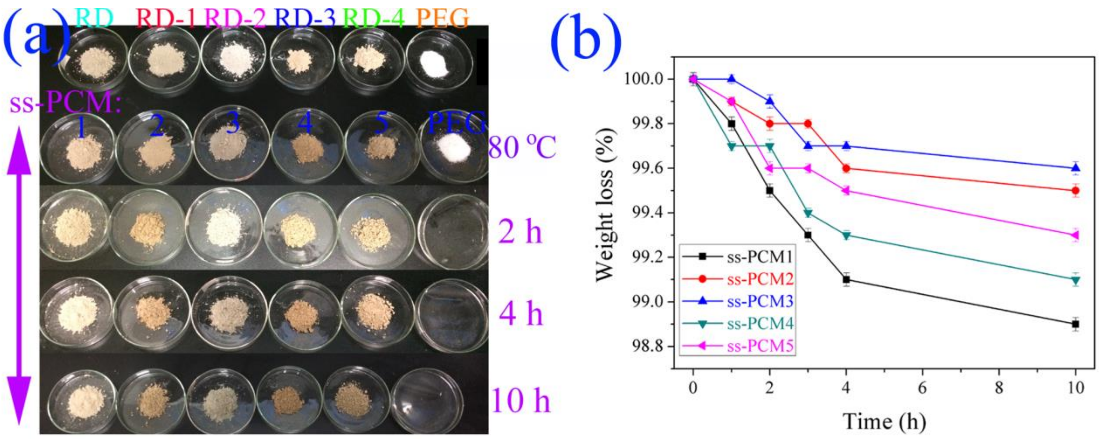

Fig. S3 (a) Photographs of modified diatomite, ss-PCMs and PEG; (b) The mass loss of the prepared ss-PCMs during the melting process

References

[1] Koruniv Z. ReviewDiatomaceous earths, a group of natural insecticides. J. *Stored Prod. Res.* **34**, 87–97 (1998).

[2] Qian T. T., Li J. H., Ma H. W., Yang J. The preparation of a green shape-stabilized composite phase change material of polyethylene glycol/SiO_2 with enhanced thermal performance based on oil shale ash via temperature-assisted sol–gel method. *Sol. Energy Mater. Sol. C.*, **132**, 29–39 (2015).
